# Protein Carbonylation as a Biomarker of Oxidative Stress and a Therapeutic Target in Neonatal Brain Damage

**DOI:** 10.3390/antiox12101839

**Published:** 2023-10-10

**Authors:** José Martínez-Orgado, María Martínez-Vega, Laura Silva, Angela Romero, María de Hoz-Rivera, María Villa, Aarón del Pozo

**Affiliations:** 1 Biomedical Research Foundation, Hospital Clínico San Carlos—IdISSC, 28040 Madrid, Spain; martinezvegamaria@hotmail.com (M.M.-V.); laura.sc95@gmail.com (L.S.); angromsan98@gmail.com (A.R.); mdehoz@ucm.es (M.d.H.-R.); m.villac.22@gmail.com (M.V.); aaron.moliner@gmail.com (A.d.P.); 2 Department of Neonatology, Hospital Clínico San Carlos—IdISSC, 28040 Madrid, Spain

**Keywords:** oxidative stress, protein carbonylation, cannabidiol, brain damage, newborn, translational models

## Abstract

Oxidative stress (OS) constitutes a pivotal factor within the mechanisms underlying brain damage, for which the immature brain is particularly vulnerable. This vulnerability is caused by the abundance of immature oligodendrocytes in the immature brain, which are highly susceptible to OS-induced harm. Consequently, any injurious process involving OS within the immature brain can lead to long-term myelination impairment. Among the detrimental repercussions of OS, protein carbonylation stands out as a prominently deleterious consequence. Noteworthy elevation of protein carbonylation is observable across diverse models of neonatal brain injury, following both diffuse and focal hypoxic–ischemic insults, as well as intraventricular hemorrhage, in diverse animal species encompassing rodents and larger mammals, and at varying stages of brain development. In the immature brain, protein carbonylation manifests as a byproduct of reactive nitrogen species, bearing profound implications for cell injury, particularly in terms of inflammation amplification. Moreover, protein carbonylation appears as a therapeutic target for mitigating neonatal brain damage. The administration of a potent antioxidant, such as cannabidiol, yields substantial neuroprotective effects. These encompass the reduction in cerebral damage, restoration of neurobehavioral performance, and preservation of physiological myelination. Such effects are linked to the modulation of protein carbonylation. The assessment of protein carbonylation emerges as a reliable method for comprehending the intricate mechanisms underpinning damage and neuroprotection within neonatal brain injury.

## 1. Introduction

Neonatal-acquired brain damage is a prevalent condition affecting 1–4 per 1000 newborns [1,2]. This encompasses a spectrum of injuries, including diffuse or focal hypoxic–ischemic damage, complications arising from prematurity, as well as damage attributed to infectious diseases or central nervous system trauma [1,2]. Each year, over two million infants globally either die or survive with permanent disabling repercussions due to such injuries [2]. Despite the pressing significance of this issue, a substantive treatment for acquired brain damage in newborns remains elusive, barring therapeutic hypothermia, which benefits approximately 60% of full-term newborns with moderate to severe hypoxic-ischemic encephalopathy [1].

The inefficacy of therapeutic strategies in the neonatal population is closely tethered to the impossibility to predict the development of brain damage after a perinatal insult in the majority of cases, as well as to the intricate pathophysiology of brain damage [1,3]. Similar to other developmental stages, the foremost drivers of brain damage comprise neuroinflammation, excitotoxicity, and oxidative stress (OS), each mutually potentiated [3,4]. Notably, the role of OS is assuming prominence due to its pivotal involvement in both structural and functional impairment.

Reactive oxygen species (ROSs), primarily originating from compromised mitochondrial respiration; along with reactive nitrogen species (RNSs), primarily generated through excessive nitric oxide (NO) production; and reactive halogen species, produced after the oxidation of halogen ions by myeloperoxidase, adversely affect protein, lipid, and DNA integrity [5,6,7,8]. These agents disrupt mitochondrial energy generation and perturb the redox state irreversibly, precipitating towards an imbalance termed OS [5,6,7]. Finally, OS can lead to endoplasmic reticulum (ER) stress [9]. Since the normal functioning of the ER requires strict control of the redox environment, the surge in OS leads to functional alteration of the ER, with the consequent impairment of normal protein synthesis and activation of a variety of signaling pathways that lead to brain damage [9].

**Figure 1:**
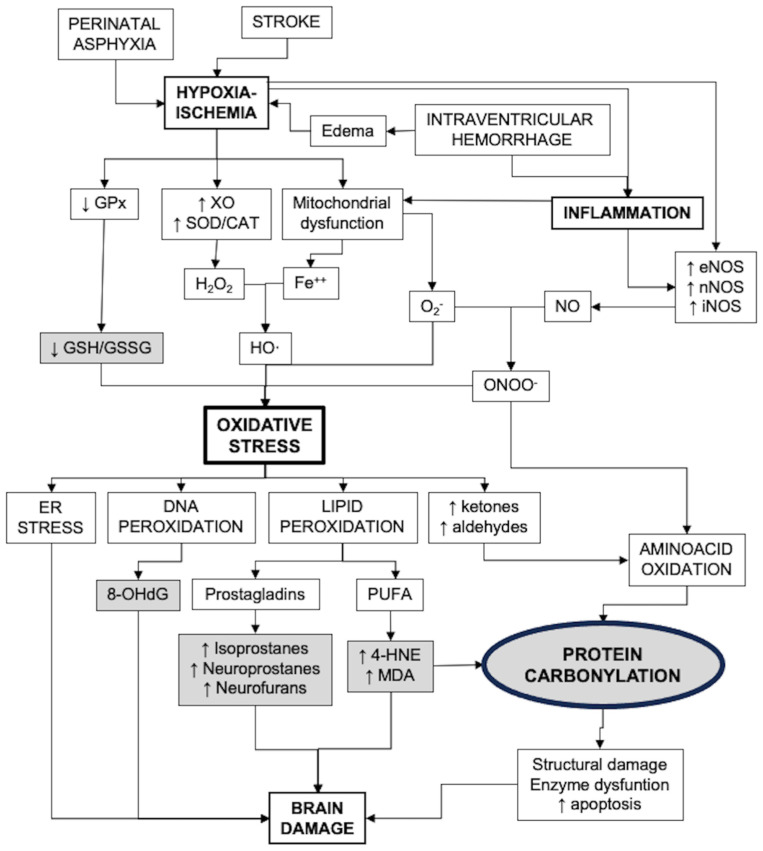
Factors involved in the development of oxidative stress after brain damage in newborns and the consequences. Strategic biomarkers are shown in gray shading. CAT: catalase; ER: endoplasmic reticulum; GPx: glutathione peroxidases; GSH: reduced glutathione; GSSG: glutathione bisulfide; MDA: malondialdehyde; 4-HNE: 4-hydroxynonenal; NOS: nitric oxide synthase; 8-OHdG: 8-hydroxy-deoxyguanosine; PUFA: poli-unsaturated fatty acids; SOD: superoxide dismutase; XO: xanthin oxidase.

Figure 1 presents a diagram that includes the main pathways of oxidative stress generation and its consequences, including biomarkers of strategic value for the study of OS in the neonatal brain.

## 2. Vulnerability of the Immature Brain to Oxidative Stress

The immature brain is notably susceptible to OS for multiple reasons. Firstly, the prenatal environment is characterized as a physiologically hypoxic environment, which results in underdeveloped antioxidant systems at birth, particularly evident in preterm newborns [5,6,7,10]. Moreover, the immature brain shows a heightened rate of oxygen consumption, a surplus of unsaturated fatty acids, and an elevated concentration of metals that catalyze reactive species formation [5,6,7,10]. Consequently, the disparity between reactive species production and antioxidant defenses is markedly accentuated in comparison to the mature brain [5,6,7,10]. Furthermore, the immature brain is highly predisposed to pathophysiological processes that augment OS. Notably, the greater density and heightened sensitivity of NMDA receptors elicit a more pronounced and deleterious response to extracellular glutamate [4,7,10]. Since glutamate reuptake enzymes are ATP-dependent, energy failure in an episode of hypoxia–ischemia leads to the extracellular accumulation of this excitotoxic neurotransmitter [4,7,10]. In fact, excitotoxicity, as an early and potent instigator, sets the stage for subsequent OS, giving rise to the concept of “excito-oxidative damage” [4]. Likewise, the immature brain is particularly susceptible to inflammatory processes, which further exacerbate OS [4,11].

A particular vulnerability of the immature brain is that, especially in the case of preterm newborns, the largest proportion of oligodendroglial cells, which will be responsible for the subsequent myelination process, are oligodendroglial precursors [12]. Among the cells constituting the oligodendroglial lineage, oligodendroglial precursors demonstrate the utmost sensitivity to OS [5,12,13]. This susceptibility arises chiefly due to their elevated iron content and heightened metabolic activity [5,12,13]. Notably, the heightened vulnerability of immature oligodendroglial cells to OS bestows upon the immature brain a distinct attribute. Unlike the adult brain, acute hypoxic–ischemic insults result in enduring myelination impairment, as observed in preclinical models of hypoxic–ischemic damage in neonatal rats [13].

Despite this vulnerability, the immature brain during the fetal period is exposed to multiple agents that can increase OS, such as environmental pollutants, infections or exposure to alcohol or other drugs [9,14,15,16]. Heavy metals, xeno-hormones, fertilizers, pesticides and herbicides are increasingly present in modern life and can reach the fetus through the mother, directly or indirectly generating an increase in OS [14]. Prenatal infections increase the inflammatory status in the fetus, either directly by contaminating the fetus itself or indirectly through the systemic inflammatory response that occurs in the mother [16]. As discussed later, inflammation is an important inducer of OS [7], including the impairment of mitochondrial function, which is an important cause of OS [16]. Among the multiple consequences of prenatal exposure to alcohol is the increase in OS, since alcohol downregulates antioxidant enzymes such as glutathione peroxidase and upregulates hydrogen peroxidase [15]. Additionally, alcohol induces an inflammatory response in the immature brain through activation of microglia and alteration of astrocyte function [15]. Prenatal drug exposure may also result in increased OS [9]. Cocaine and methamphetamine may alter mitochondrial function and increase brain dopamine levels, leading to increased production of free radicals by auto-oxidation and metabolic oxidation by the action of monoamine oxidase [9]. Opioids increase lipid peroxidation and downregulate glutathione peroxidase to reduce brain glutathione concentration [9]. Nicotine, in addition to all these actions, is a strong and important source of nitric oxide (NO) [9], which, as will be discussed below, is also a damaging agent per se and because when produced in extraordinary quantities is one of the main agents causing protein carbonylation, which is one of the most serious consequences of OS [5,17].

In the present review we will focus on highly prevalent perinatal-acquired brain pathology in which oxidative stress plays an important role, such as diffuse or focal hypoxic–ischemic damage or the cerebral consequences of prematurity [1,2]. Furthermore, sick newborns are frequently exposed to episodes of hypoxia/hyperoxia, ischemia or infection, all of them leading to increased OS [5].

Thus, it is clear that OS plays a particularly prominent role in the process of brain damage in newborns [7].

## 3. Assessment of Oxidative Stress in Neonatal Brain Damage

There are different ways to approach the evaluation of OS in studies on brain damage.

One of them is the assessment of the activity of enzymes related to antioxidant activity, specifically the quantification of the expression of superoxide dismutase, catalase and glutathione peroxidase [6,7,10]. The expression of catalase and superoxide dismutase is elevated after acute neonatal brain damage in response to increased OS, and there is a correlation between said increase in expression and the severity of the damage [6,10]. However, in the case of glutathione peroxidase, OS usually leads to a decrease in its expression, resulting in a deficiency in reduced glutathione (GSH), which is one of the main antioxidants in the neonatal period [7]. Thus, the study of the glutathione pathway is of special importance in neonatal pathology. This not only includes the quantification of the expression of the enzyme glutathione peroxidase but also that of the reduced glutathione/glutathione disulfide (GSH/GSSG) ratio, which is a very precise marker of OS shown to be elevated both in experimental models and in patients with hypoxic–ischemic damage [7].

As previously mentioned, lipid peroxidation is one of the most relevant consequences of OS and one of the factors that participate in OS spreading [6,7,10]. One of the most classic markers of lipid peroxidation is malondialdehyde (MDA), a product of the oxidation of n-3 and n-6 fatty acids [6]. The ease of its determination by an available spectrophotometric technique known as a thiobarbituric acid assay, together with the fact that MDA is soluble in water so it can be determined in urine in a non-invasive way, has made it very popular in studies of OS [6,7,10]. However, MDA is not very specific for brain damage [6]. More precise markers of brain lipid peroxidation are related to the oxidation of prostaglandins, specifically the determination of isoprostanes, neuroprostanes and neurofurans by gas chromatography coupled with mass spectrometry coupled with liquid chromatography (LC-MS/MS) [6,7,10]. However, although the levels of F2-isoprostanes reflect with great fidelity the intensity of OS, they have not shown a correlation with the severity of brain damage in newborns [6]. A relatively recent marker of lipid peroxidation is 4-hydroxynonenal (4-HNE), a product of n-6 fatty acid oxidation [6,7,8,10]. Due to its high cytotoxicity and constant presence in all conditions related with OS, it is now considered a key biomarker of lipid peroxidation [8]. In addition, the possibility of detecting 4’HNE in tissues by immunohistochemistry studies makes 4-HNE a useful biomarker to assess the geographical distribution of OS in affected organs [8]. Although its concentration is elevated in cord blood of newborns with perinatal distress, there is very little evidence of in its use in human newborns [6]. However, in experimental models of hypoxic–ischemic brain damage in newborn rats, increased levels of 4-HNE have been described in relation to OS and the severity of apoptotic death processes [18]. C4-HNE is an OS biomarker also of special interest because, as will be discussed later, it participates in the protein oxidation process [19].

Along with lipid peroxidation, the assessment of DNA peroxidation is another method of quantifying the severity of OS and its consequences for inducing brain damage [6,7,10]. The most popular marker for this purpose is the oxidized DNA nucleoside 8-hydroxy deoxyguanosine (8-OHdG) [6,7,10]. 8-OHdG is a reliable marker of DNA damage, but it is not specific for hypoxic–ischemic damage since it appears elevated in other neonatal pathologies such as epileptiform encephalopathies or infections, and for now a relationship with the severity of the neurological damage has not been demonstrated [6].

In this review we will focus on another way of assessing OS: the quantification of protein oxidation.

## 4. Protein Carbonylation and Oxidative Stress

Protein oxidation, due to its potential to disrupt the functionality of multiple proteins, plays a considerably detrimental role by perturbing the cellular redox balance and influencing the cell cycle, ultimately culminating in neuronal demise [8,17]. Among the characteristic consequences of OS, protein carbonylation emerges as an irrevocable modification, imparting notable damage [17,19]. Protein carbonylation involves the direct oxidation of lysine, arginine, proline, and threonine side chains, resulting in the formation of reactive ketones or aldehydes that subsequently react with 2,4-dinitrophenylhydrazine (DNPH) to yield hydrazones [19]. Carbonylated proteins are primarily degraded by the 20S proteasome, but after massive production carbonylated proteins, additional poorly understood mechanisms of decarbonylation are initiated, including thiol-dependent reduction and pyridoxamine scavenging [8]. When these systems are overwhelmed, carbonylated proteins accumulate in the cell, exerting cytotoxic effects [8]. Beyond mere structural and functional changes, protein carbonylation heightens cellular susceptibility to apoptotic triggers like calpains [17]. Consequently, protein carbonylation has garnered significance as an imperative marker of OS, not solely due to its structural and functional ramifications, but also due to its sensitivity and specificity [6,7,8,10].

A common difficulty in using protein carbonylation for research or diagnostic purposes is that, while LC-MS/MS is the established approach for detecting and quantifying oxidative markers, including carbonylated proteins, its extensive cost and requirement for substantial expertise constrain its widespread adoption [6]. To overcome this limitation, alternative methodologies have been developed to offer more accessible techniques in terms of both methodology and expenditure [6,10]. One of these methodologies is the Oxyblot technique, which was extensively used in our experiments (Oxyblot^TM^, Merck Millipore, Merck KGaA, Darmstadt, Germany) [20,21,22,23]. Oxyblot is a commercially available Western blot method underpinned by anti-DNPH antibodies. It effectively detects carbonylated proteins, comparable in efficiency to other methods such as spectrophotometry or ELISA [17,24]. However, its distinctive merit lies in its user-friendly and cost-effective implementation [17,24]. The detection of protein carbonylation involves enhanced chemiluminescence, with quantification performed through densitometric analysis. The quantified data are subsequently normalized by considering the total protein loading, as obtained through the Red Ponceau technique in the sample [20,21,22,23].

Protein carbonylation can arise from the action of various oxidizing agents [19]. A major source is the reactive species OH·, a product of the Fenton reaction between H_2_O_2_ and ionic metals, in particular Fe^2+^, that produces very toxic carbonyl derivatives of lysine, arginine and threonine [8]. Of special relevance are the peroxidation processes of polyunsaturated fatty acids, producing particularly toxic carbonyl species such as α,β-unsaturated aldehydes and ketoaldehydes, in particular the 4-HNE products, therefore playing a prominent role in the so-called secondary protein carbonylation processes [8,19]. Protein carbonylation can also result from non-enzymatic glycation, particularly in conditions with hyperglycemia, lysyl oxidation and polyphenol reactions [8]. Other major contributors to protein carbonylation are reactive nitrogen species (RNSs), resulting in protein nitrosylation [5,6,17]. RNSs primarily originate from the interaction of NO with other free radicals, engendering diverse reactive species, with peroxynitrite (ONOO^−^) formation being the most detrimental consequence. ONOO^−^ emerges from the union of NO and superoxide anions [5]. The synthesis of NO is facilitated by NO synthases (NOSs) [5,19]. Constitutive endothelial and neuronal NOSs (eNOS and nNOS, respectively) yield modest NO levels; conversely, inducible NOS (iNOS), once activated, triggers massive NO production through an irreversible process [5,25].

**Figure 2:**
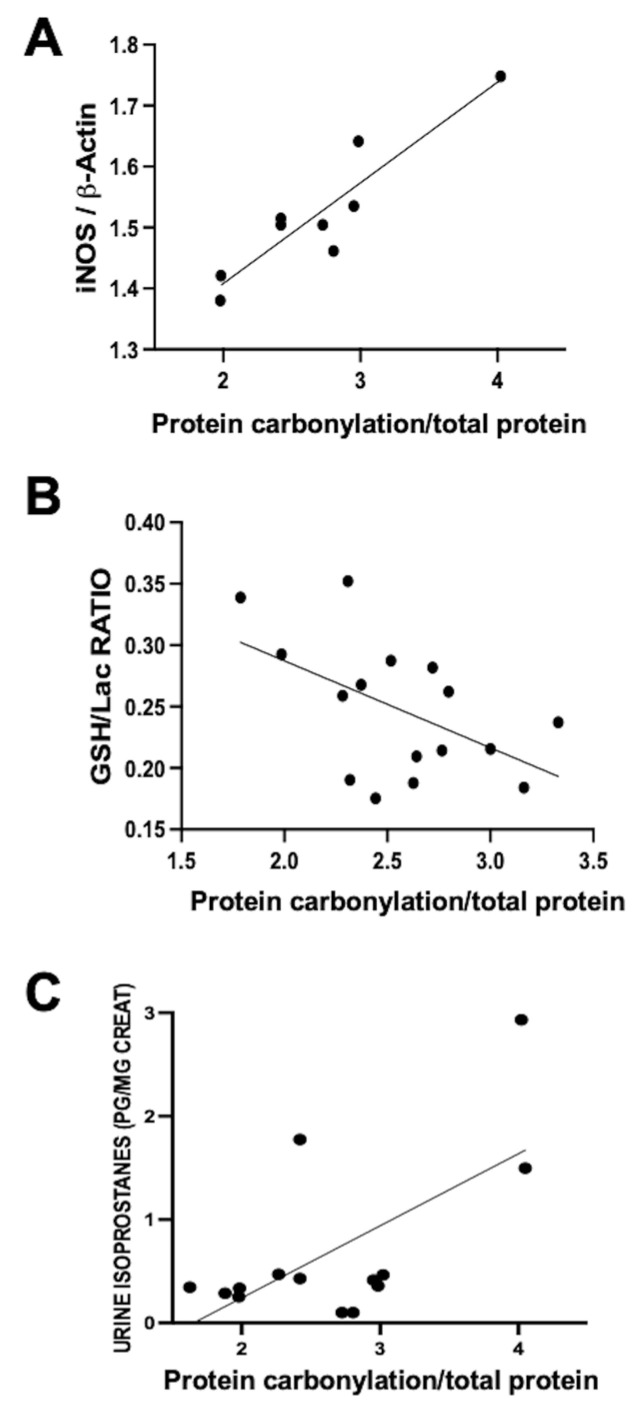
Relationship between protein carbonylation in the brain and different biomarkers of oxidative stress ((**A**,**B**): in brain samples; (**C**): in urine) in 1- to 3-day-old piglets 6 h after a hypoxic–ischemic insult (bilateral carotid artery occlusion plus exposure to FiO_2_ 10% for 30 min). Simple linear regression ((**A**): y = 0.1654 × X + 1.077, R^2^ = 0.84, *p* = 0.0005; (**B**): y = −0.07080 × X + 0.4289, R^2^ = 0.27, *p* = 0.03; (**C**): y = 0.6972 × X − 1.153, R^2^ = 0.41, *p* = 0.01). iNOS: inducible nitric oxide synthase; GSH: reduced glutathione; Lac: lactate. The figures show results from analyses carried out on data obtained from the studies described in references [20,21].

This process’s involvement was investigated by our group in a neonatal piglet model of diffuse hypoxic–ischemic brain damage. In this model, 1- to 3-day-old piglets underwent bilateral carotid blood flow interruption under hypoxic conditions (FiO_2_ 10%) for 30 min [20,21]. Using this model, a direct correlation was observed within the same parieto-temporal cortex sample between iNOS expression, assessed via Western blot, and protein carbonylation intensity, gauged using Oxyblot (Figure 2A). Nevertheless, no correlation was identified between protein carbonylation and increased cyclo-oxygenase 2 expression, a key ROS producer (y = 0.04762 × X + 0.9596, R^2^ = 0.02, *p* = 0.59, where y= COX-2 expression normalized to ß-actin and X = protein carbonylation). This data underscores the paramount involvement of RNS in the protein carbonylation process.

The utility of protein carbonylation determination as an OS marker is validated in studies carried out using the data from the aforementioned hypoxic–ischemic damage model in neonatal piglets [20,21]. Alongside protein carbonylation, lipid oxidation is a consequential outcome of OS [5,6,7,10]. As previously described, quantifying urine isoprostane concentration, a maker of lipid oxidation, reliably reflects OS [6,7,10]. In hypoxic–ischemic neonatal piglets, a direct correlation emerged between urine isoprostane concentration and protein carbonylation severity in brain samples (Figure 2B). These findings affirm the close interconnection between lipid oxidation and protein carbonylation [19]. Glutathione constitutes a pivotal element of the natural antioxidant defenses; hence, the decline in GSH content in tissues is a classic OS biomarker [7,10]. In hypoxic–ischemic neonatal piglets, a direct relationship between protein carbonylation severity and GSH content reduction within the same brain samples was established (Figure 2C). Collectively, these results indicate that protein carbonylation transcends being merely a marker of protein oxidation; rather, it steadfastly signifies OS at large [19].

## 5. Protein Carbonylation in Newborn Brain Damage

**Figure 3:**
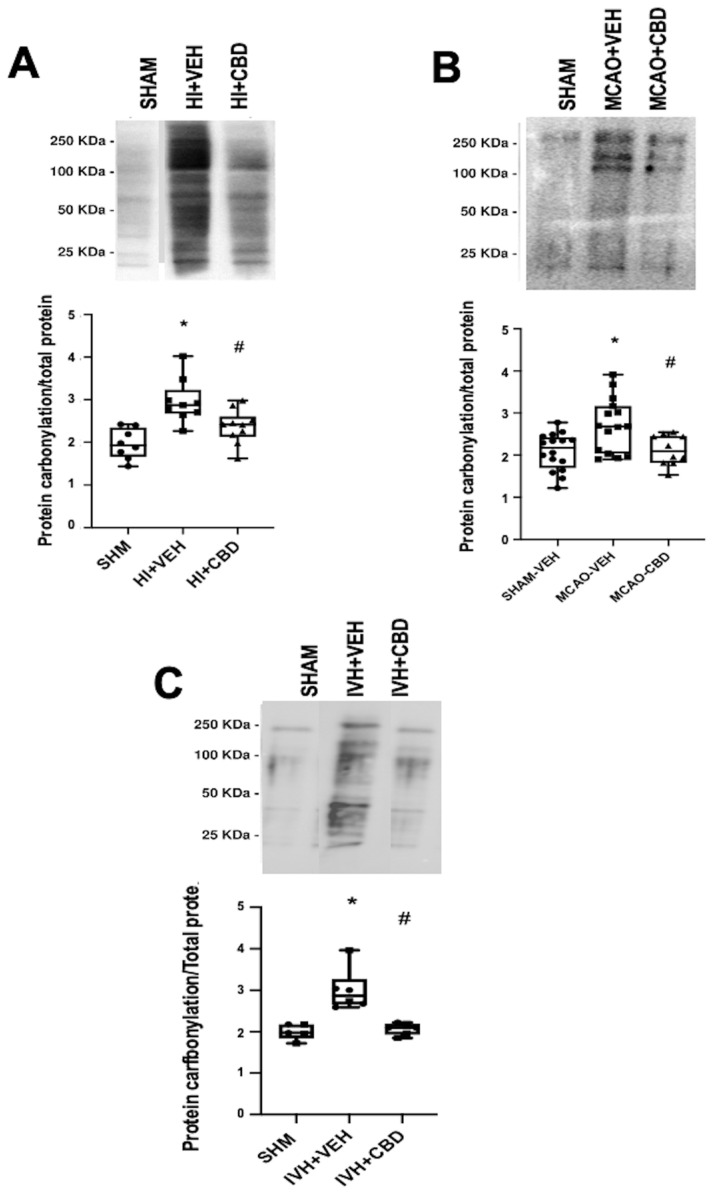
Representative Oxyblot films assessing protein carbonylation, and the corresponding graphical representation of the densitometric analysis, studied in brain samples obtained (**A**) 6 h after a hypoxic–ischemic (HI) insult (bilateral carotid artery occlusion plus exposure to FiO_2_ 10% for 30 min) to 1- to 3-day-old piglets. (**B**) Seven days after stroke induction by reversible middle cerebral artery occlusion (MCAO) in 7- to 8-day-old Wistar rats; (**C**) five days after induction of intraventricular hemorrhage (IVH) by paraventricular injection of Chlostridium collagenase in 1-day-old rats. Injured animals were treated post insult with vehicle (VEH) or cannabidiol (CBD). Non-injured animals served as controls (SHAM). Boxes represent the median and interquartile range, and the whiskers are the maximum and minimum values. Dots represent individual data. (*) *p* < 0.05 vs. SHAM, (#) *p* < 0.05 vs. VEH, all analyzed by Kruskal–Wallis with Dunn’s test for multiple comparisons. The figures show the results from analyses carried out on data obtained from the studies described in references [20,21,22,29].

As previously stated, our investigations have confirmed the elevation in protein carbonylation as a consequence of diffuse hypoxic–ischemic damage within brain cortex samples from asphyxiated neonatal piglets (Figure 3A) [20,21]. This model has allowed us to identify the pivotal involvement of protein carbonylation in precipitating brain damage. In this context, we employed proton nuclear magnetic resonance spectroscopy (^1^H-NMR) to quantify the lactate/N-acetyl aspartate (Lac/NAA) ratio in brain cortex samples [21]. The Lac/NAA ratio is a widely adopted indicator of brain damage; it reflects the increase in lactate concentration due to compromised aerobic mitochondrial function coupled with a decrease in N-acetyl aspartate concentration resulting from a reduced neuronal population [26]. The elevated Lac/NAA ratio corresponds proportionally to the severity of brain damage [26]. Remarkably, a direct correlation was identified between the extent of protein carbonylation and the rise in the lactate/N-acetyl aspartate ratio in asphyxiated piglets (y = 0.8586 × X + 1.034, R^2^ = 0.51, *p* = 0.004; where y = Lac/NAA ratio and X = protein carbonylation).

The importance of the role of protein carbonylation in the induction of neonatal brain damage after a hypoxic–ischemic insult is highlighted by the fact that neurons from the most vulnerable areas of the neonatal brain to hypoxia–ischemia are precisely those that present higher expression levels of nNOS [27]. As mentioned above, the main source of protein carbonylation is NO [19]. Although the greatest weight of nitric oxide production after positive ischemic damage corresponds to iNOS, the overexpression of nNOS plays an important role in this process [25]. In immature rats, the expression of nNOS evolves at the end of gestation so that in the perinatal period nNOS expression is higher in the deeper layers of the cortex, in the basal ganglia, and in the CA1 area of the hippocampus, that is, the most vulnerable areas to hypoxic–ischemic damage [27].

Notably, the utility of detecting and quantifying protein carbonylation is not restricted to hypoxic–ischemic damage but extends to diverse brain pathologies involving oxidative stress [12]. In this context, we examined protein carbonylation following focal ischemic insult in an acute ischemic stroke model in neonatal rats [3]. This model is an adaptation of the temporal middle cerebral artery occlusion (MCAO) adult model [28]. To induce acute ischemic stroke, reversible MCAO was induced by introducing an occlusion through the left common carotid artery to block MCA blood flow for 3 h in 7- to 9-day-old Wistar rats, yielding a consistent and uniform cortico-striatal infarct [29]. The study of the brain cortex area adjacent to the infarct in frozen samples [29] exhibited a marked elevation in protein carbonylation (Figure 3B).

Moreover, we delved into the role of protein carbonylation in a different form of neonatal-acquired brain damage, specifically, intraventricular hemorrhage (IVH)-induced damage in very immature brains [22]. This model is induced by Chlostridium collagenase injection in the region adjacent to the left Germinal Matrix in 1-day-old Wistar rats, mirroring the brain developmental stage akin to preterm newborns at 24–26 weeks of gestation [30]. Remarkably, an increase in protein carbonylation was also evident in ipsilateral striatum from frozen brain samples obtained from that model (Figure 3C). The IVH model is particularly insightful for studying OS due to the amalgamation of factors contributing to OS, i.e., mitochondrial dysfunction, hyperproduction of nitric oxide, and a profusion of free radicals stemming from reperfusion, alongside extravasated blood carrying high iron and other oxidant metal content [31]. Thus, OS emerges as a pivotal player in IVH-induced damage within highly immature brains [31,32,33]. Pertinently, a noteworthy facet concerning OS, and by extension protein carbonylation, is the fact that the particular vulnerability of the immature brain due to the addition of unfavorable factors can determine that OS was disproportionately high compared to the apparent severity of the insult [5,7]. For instance, in the IVH model in neonatal rats, no direct correlation was evident between protein carbonylation and the volume of IVH-induced brain damage assessed through magnetic resonance imaging (y = −0.02952 × X + 27.24, R^2^ = 0.008, *p* = 0.76; where y = protein carbonylation and X = volume of brain damage as % brain volume). The lack of a direct relationship between the extension of brain damage and subsequent OS development potentially elucidates the proclivity of even minor IVH in extremely preterm newborns to evoke enduring neurodevelopmental impairment [34].

## 6. Protein Carbonylation and Neuroinflammation

Excitotoxicity and oxidative stress are subsequent processes [4]. Excitotoxicity, particularly the surge in glutamate release and excessive activation of glutamate receptors, is an early event following cerebral insult, inciting subsequent cellular responses, including OS [4]. Consequently, the peak in excitotoxicity typically precedes that of OS [4,7]. Thus, in frozen brain samples obtained using the asphyxiated piglet model [21], concurrent assessment of protein carbonylation via Oxyblot and glutamate concentration via ^1^H-NMR within the same brain samples unveiled no discernible correlation (y = 0.003904 × X + 0.4757, R^2^ = 0.02, *p* = 0.8 t; where y = glutamate/NAA ratio and X = protein carbonylation).

**Figure 4:**
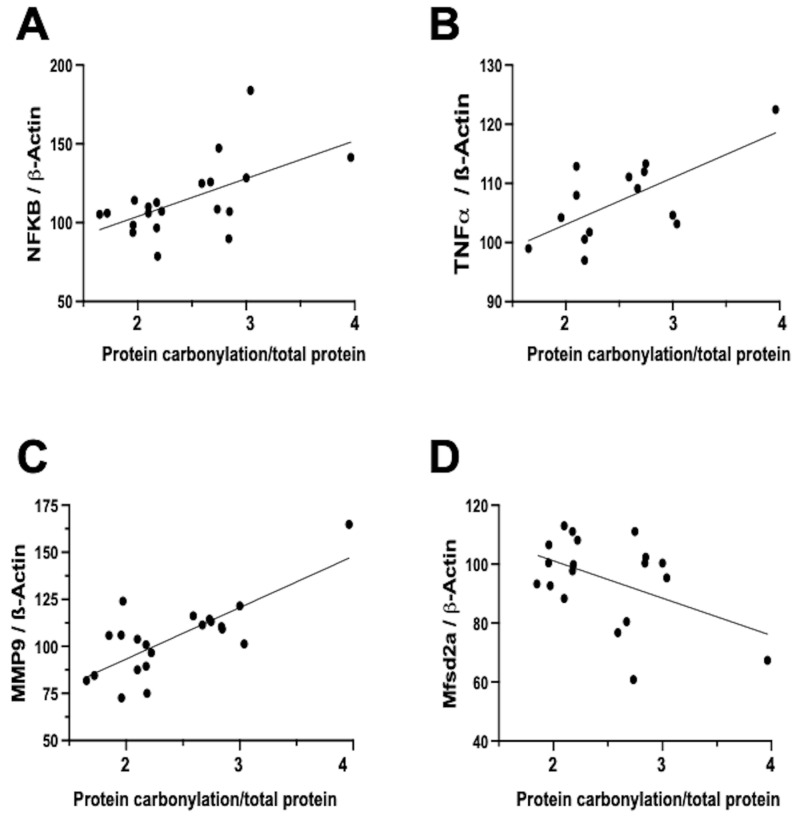
Relationship between protein carbonylation determined using Oxyblot and biomarkers of inflammation and blood–brain barrier integrity assessed using Western blot in brain samples obtained five days after induction of intraventricular hemorrhage (IVH) by paraventricular injection of Chlostridium collagenase in 1-day-old rats. Simple linear regression ((**A**): y = 0.4809 × X + 55.84, R^2^ = 0.33, *p* = 0.008; (**B**): y = 0.1577 × X + 87.29, R^2^ = 0.44, *p* = 0.009; (**C**): y = 0.5466 × X + 38.61, R^2^ = 0.57, *p* < 0.0001; (**D**): y = −0.2535 × X + 126.5, R^2^ = 0.22, *p* = 0.04). NFkB: nuclear factor k B; TNFa: tumor necrosis factor a; MMP-9: matrix metalloproteinase 9; Mfsd2a: major facilitator superfamily domain-containing protein 2a. The figures show results from analyses carried out on data obtained from the study described in reference [29].

In contrast, the relationship between neuroinflammation and OS is notably more direct and temporally aligned [7]. Firstly, inflammation activates different signaling pathways that determine the dysfunction of the mitochondria, particularly in relation to oxidative phosphorylation, and therefore impairs the mitochondrial modulation of the redox state [16]. In this way, inflammation is a direct cause of oxidative stress. In addition, a prominent contributor to the instigation, perpetuation, and amplification of OS is the infiltration of brain tissue by inflammatory cells [5,7,32]. Biochemical studies on frozen brain samples obtained from the neonatal rat model of IVH substantiates a direct association between the severity of protein carbonylation and heightened NFκB expression (Figure 4A). NFκB activation triggers augmented cytokine production [22,35], leading us to validate a direct correlation between the extent of protein carbonylation and elevated TNFα concentration in the brain (Figure 4B). Additionally, NFκB activation is intrinsically linked to the initiation of apoptotic pathways [36], thus rendering NFκB activation pivotal in eliciting neuronal demise subsequent to acute insults in immature brains [11].

Notably, inflammation activated by OS is not solely mediated via the NFκB pathway. In our neonatal rat IVH model, we ascertained a direct link between the degree of protein carbonylation and elevated matrix metalloproteinase 9 (MMP-9) expression (Figure 4C). While NFκB does contribute to MMP-9 activation, it predominantly arises from the activation of inflammatory cells, instigating extracellular matrix degradation [7,37]. This process not only plays a role in post-insult brain damage, including white matter injury, but also contributes to blood–brain barrier (BBB) structural impairment [37]. Remarkably, protein carbonylation intensity as assessed in frozen samples obtained in the IVH model correlated with heightened MMP-9 expression, thus influencing BBB structural alterations. Additionally, the severity of protein carbonylation in this model corresponded to diminished expression of major facilitator superfamily domain-containing protein 2A (Mfsd2a) (Figure 4D), a sensitive marker of BBB transcellular activity impairment [33]. Consequently, in neonatal rats following IVH, OS is not only associated with BBB structural damage but also functional BBB impairment. This holds particular significance as increased BBB permeability is linked to escalated infiltration of inflammatory cells [22], further amplifying OS in a detrimental vicious circle.

## 7. Protein Carbonylation as a Target for Neuroprotection in Neonatal Brain Damage

The modulation of carbonylated protein production presents a promising alternative approach compared to conventional strategies focusing solely on free radical scavenging, as explored in preclinical models of carbonyl-associated disorders such as diabetes [19].

A variety of strategies have been tested to reduce OS as part of neuroprotective treatment in newborns with hypoxic–ischemic brain damage, in particular, chelators such as allopurinol, scavengers such as melatonin, lipid peroxidation inhibitors such as vitamin E, reducing agents such as erythropoietin, or even treatments with a non-specific antioxidant effect such as indomethacin or hypothermia [5]. None of these treatments have shown convincingly positive effects in reducing OS with any appreciable therapeutic effects [5]. In relation to the prevention of the harmful effects on brain development of prenatal toxic substances, such as alcohol, the increase in maternal intake during pregnancy of vitamin E or C, beta-carotene, folic acid or omega-3 fatty acids has shown positive effects that would justify its widespread use [15]. In addition, drugs such as astaxanthin or EUK-134 have shown very promising effects in preclinical models of fetal brain toxicity due to alcohol [15].

Our research group has dedicated extensive efforts to investigate the protective potential of cannabidiol (CBD) in various models of neonatal brain damage [3]. CBD, a non-psychotropic component of *Cannabis sativa*, exhibits considerable efficacy in diminishing brain damage assessed through neuroimaging, histological, and biochemical studies following diffuse or focal hypoxic–ischemic insults in neonatal rodents and piglets, ultimately restoring long-term functional performance [3]. CBD functions as a potent antioxidant due to its inherent molecular structure [38,39,40]. In vitro analyses have shown that CBD’s antioxidant effectiveness surpasses that of other robust antioxidants like ascorbate or tocopherol [40]. Furthermore, CBD can stimulate antioxidant effects by activating nuclear erythroid 2-related factor (Nrf2) and influencing the expression and activity of antioxidant enzymes such as superoxide dismutase and glutathione peroxidase [39]. Furthermore, CBD modulates factors that contribute to OS post-brain insults, including inflammation and excitotoxicity [3], particularly pertinent in the context of neonatal brain damage [3].

In neonatal piglets subjected to hypoxic–ischemic injury, a single administration of CBD at 1 mg/kg, given 30 min after the insult [21], effectively prevents the surge in protein carbonylation (Figure 3A). Similar prevention of increased protein carbonylation is observed in brain samples obtained following CBD treatment at 1 mg/kg administered 10 min after MCAO induction (Figure 3B) or 5 mg/kg administered 6 h after intraventricular hemorrhage (IVH) initiation in neonatal rats (Figure 3C). This robust antioxidant effect aligns with substantial neuroprotective outcomes [21,29].

As previously elucidated, immature oligodendroglial cells are particularly susceptible to OS. Thus, the potent antioxidant properties of CBD could account for its ability to prevent medium- and long-term myelination impairment in seven-day-old rats exposed to diffuse hypoxic–ischemic insults following 10% oxygen exposure for two hours, after electrocoagulation of the left carotid artery [13]. Hypoxic–ischemic neonatal rats exhibit diminished global myelin synthesis, axon count, and myelin sheath thickness in the cortex and external capsule one-month post-insult, attributed to oligodendroglial precursor maturation disruption [13]. Arrested oligodendroglial maturation is the key factor leading to long-term white matter injury in immature brain, which is the key substrate for long-lasting invalidating sequelae such as cerebral palsy [41]. Notably, CBD administration at 1 mg/kg, applied 30 min post-insult, safeguards the oligodendrocyte maturation process, consequently restoring normal myelin synthesis, axon count, and myelin sheath thickness [13]. These findings underscore the remarkable efficacy of antioxidant treatment resulting in the prevention of protein carbonylation, capable of not only mitigating immediate post-insult brain damage but also averting the emergence of enduring sequelae.

## 8. Conclusions

Protein carbonylation emerges as a pivotal facet of OS within the central nervous system, offering a robust, dependable, and cost-effective means of OS assessment. Its significance is notably underscored in the investigation of neonatal brain damage, where the immature brain is particularly susceptible to the broader impacts of OS, with protein carbonylation taking center stage. The presence of escalated protein carbonylation across diverse models of diffuse or focal hypoxic–ischemic brain injury, as well as intraventricular hemorrhage, spanning various developmental stages and species, including rodents and larger mammals, substantiates its ubiquity in neonatal brain damage.

Considering the potential therapeutic targeting of protein carbonylation for a spectrum of carbonyl-associated conditions, it also emerges as a promising avenue for neonatal brain damage treatment. CBD, a potent antioxidant, demonstrates compelling neuroprotective effects intertwined with the modulation of protein carbonylation. As such, the evaluation of protein carbonylation takes on paramount importance as a tool for delving into the intricate mechanisms underpinning both damage and neuroprotection within neonatal brain injuries.

## Data Availability

The datasets generated during and/or analyzed in this study are available from the corresponding author on reasonable request.
